# Isolation, Toxigenic Potential, and Mating Type of *Fusarium pseudograminearum* Causing Wheat Crown Rot in Hebei, China

**DOI:** 10.3390/jof11120844

**Published:** 2025-11-28

**Authors:** Jianzhou Zhang, Wenyu Wang, Jianhua Wang, Jiahui Zhang, Hao Li, Baizhu Chen, Chunying Li

**Affiliations:** 1 Wheat Research Institute, Henan Academy of Agricultural Sciences, Zhengzhou 450002, China; zhangjianzhou00@163.com (J.Z.); lichunying0661@163.com (C.L.); 2 Institute for Agro-Food Standards and Testing Technology, Shanghai Academy of Agricultural Sciences, Shanghai 201403, China; m230100125@st.shou.edu.cn (W.W.); jiahuizhang1224@163.com (J.Z.); m240100147@st.shou.edu.cn (H.L.); m240100146@st.shou.edu.cn (B.C.)

**Keywords:** fusarium crown rot, *Fusarium pseudograminearum*, trichothecene genotypes, mating type

## Abstract

Fusarium crown rot (FCR) is a devastating fungal disease of wheat in China that causes substantial yield losses and deterioration of grain quality. To clarify the pathogen composition and associated mycotoxin risks of FCR in Hebei Province, a comprehensive field survey was conducted during the critical growth stage from flowering to maturity (April to May) of the 2024 wheat season from 46 sites. Fungal isolates were obtained from symptomatic wheat stem bases and were identified through morphological and molecular analyses. In total, 156 *Fusarium* isolates were obtained, and from these isolates, 12 *Fusarium* species were identified based on species-specific PCR and DNA sequencing of the *translation elongation factor 1-α* (*TEF1*) loci. Of these *Fusarium* isolates, 118 were identified as *Fusarium pseudograminearum*, 16 identified as *F. graminearum* and the remaining isolates consisted of *F. acuminatum*, *F. asiaticum*, *F. boothii*, *F. culmorum*, *F. equiseti*, *F. flocciferum*, *F. incarnatum*, *F. proliferatum*, *F. sinensis*, and *F. verticillioides*. The results revealed that *F. pseudograminearum* with the 15ADON genotype was the predominant species, accounting for 75.64% of all the isolates, followed by *F. graminearum*. Trichothecene genotyping revealed that 91.53% of the *F. pseudograminearum* strains possessed the 15ADON genotype (108 isolates), while 8.47% exhibited the 3ADON genotype (10 isolates). Although differences were observed within *F. pseudograminearum* in *MAT1-1* and *MAT1-2* distributions among different sampling regions, a well-balanced mating type ratio was identified across Hebei Province. Population genetic analysis based on composite genotypes (trichothecene and mating type) revealed moderate to high genetic diversity within the *F. pseudograminearum* population. Recent studies on causal *Fusarium* species, trichothecene genotypes, and their distribution in China are compared and discussed. These findings may have implications in managing this significant fungal disease.

## 1. Introduction

Wheat is a critical staple crop for global food security. Human diets and animal feeds rely largely on wheat and related products as the primary source of calories and proteins [1]. In China, it serves as a primary food source for a large proportion of the population (accounting for 45% to 50% of the national total population), making its stable production vitally important [2]. However, wheat yields are consistently threatened by multiple biotic stresses, among which, Fusarium crown rot (FCR) has become a devastating soil-borne disease worldwide as it causes severe yield losses and quality deterioration [3]. FCR infects wheat at all growth stages, and infection of the root and crown can cause constriction of the vascular system, which restricts the absorption and transfer of water and nutrition. The typical symptoms of the disease include necrosis and browning of the stem base and leaf sheaths. In severe cases, the pathogen invades the vascular tissue disrupting water movement, leading to whiteheads, impaired grain filling, and even plant death [4,5].

Fusarium crown rot is an emerging destructive and economically important disease of wheat worldwide. FCR was first observed in Australia in 1951 [6]. To date, the disease has been reported across multiple continents, including Europe, Africa, North America, and Southwest Asia, confirming its widespread geographic impact [7]. Over recent decades, FCR has led to substantial wheat yield losses worldwide. In Australia, the average annual yield losses of wheat caused by FCR are estimated at 10%, corresponding to approximately AUD 88 million in economic damage [8]. In the United States, FCR has been reported to cause an average wheat grain yield loss of 10%. Notably, FCR-induced wheat yield losses in the Pacific Northwest of the United States are routinely estimated to be as high as 35% [9]. Within China, the disease occurred with moderate to severe intensity over the last decade, and the plant infection rate reached as high as 15% in certain wheat-growing regions. The statistical results from the agricultural sector indicate that the national occurrence area of wheat FCR reached about 4 million hectares in China in 2024 [10]. Additionally, the economic impact of FCR is further exacerbated by the ability of its primary causative agents, fungi within the genus *Fusarium* spp., to produce various mycotoxins (for example trichothecenes and zearalenone) that contaminate grains, posing serious threats to human and animal health [11,12,13].

Globally, in nature, FCR of wheat is caused by a complex infection of multiple *Fusarium* species and represents a serious disease in major grain-producing regions [14]. Plenty of *Fusarium* species have been reported to cause FCR, primarily *Fusarium pseudograminearum* (Aoki, T. & O’Donnell, K), *F. culmorum*, and members of the *F. graminearum* species complex (FGSC). There are variations in the composition of *Fusarium* species causing FCR in different wheat-growing regions around the world. Among the complex of *Fusarium* spp. associated with FCR of wheat, *F. pseudograminearum* has been reported as the most frequently isolated species in Australia [15,16], North America [4,9], and China [10,17,18,19,20,21]. However, *F. culmorum* was reported as the main FCR pathogen in the UK [22], Turkey [23,24], and Algeria [25,26]. As recently concluded by Özer et al. [27], the FCR pathogen *F. culmorum* was the most frequently isolated and the most aggressive fungus among all species isolated from wheat in Azerbaijan. Among the *Fusarium* species isolated from diseased wheat samples in Central Asia, such as Kazakhstan and Kyrgyzstan, *F. acuminatum* was found to be the predominant fungal species [28,29]. According to the surveys to date, *F. pseudograminearum* is frequently reported as the predominant pathogen in warm and arid areas while *F. culmorum* is typically found in cooler, high-rainfall regions. Additionally, these *Fusarium* pathogens are also the causal agents of Fusarium head blight (FHB) of wheat around the world.

Prior to 2010, FCR existed in China but caused limited damage [30]. However, since then the occurrence of FCR has been increasing, particularly in warm and semi-arid area of China, such as the Huanghuai Plain wheat-growing region [21], and with more serious in Henan, Hebei, and Shandong provinces [10,18,19,30,31]. In the past decade, the severity of FCR is treading towards epidemic status in certain wheat-growing regions of China [30]. Due to the frequent occurrences and severe damage to wheat grain production, currently, *Fusarium*-induced crown rot of wheat has been listed as one of the four main wheat diseases in China. Information on *Fusarium* species composition is essential for designing effective management strategies, especially since different fungal pathogens exhibit varying degrees of sensitivity to different fungicides [6,19,21,32,33,34,35]. Previous surveys on agents causing FCR revealed that many *Fusarium* species were the pathogens responsible for the disease in China. However, significant differences in pathogen compositions and their mycotoxin profiles were observed due to varying temporal and spatial factors. To our knowledge, relatively few surveys on the *Fusarium* population causing FCR of wheat in Hebei Province have been reported. Building upon the foundational work of recent studies (e.g., Mawcha et al., 2025 [20]) that identified *F. pseudograminearum* as the dominant FCR pathogen in Hebei, our 2024 study addresses key knowledge gaps through expanded sampling across 46 sites. It provides the first comprehensive analysis of mating type structure in *F. pseudograminearum* in the region and presents an updated toxin genotype profile, confirming the fixation of the 15ADON genotype alongside novel reports of minor fusaria. This work establishes a critical baseline for monitoring future pathogen population dynamics changes and informs the development of targeted management strategies.

## 2. Materials and Methods

### 2.1. Sample Collection

**Figure 1:**
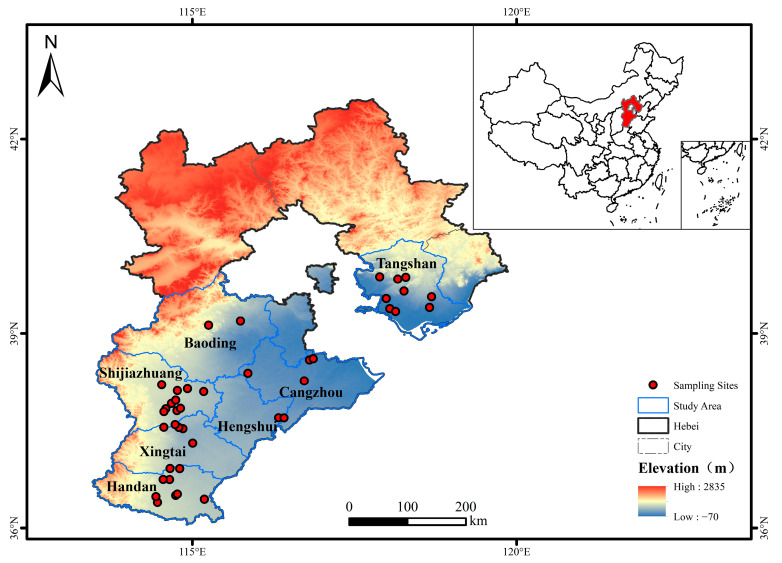
Schematic map showing the spatial distribution of sampling sites in 7 cities of Hebei Province, China, in 2024.

In 2024, a comprehensive survey was conducted during the critical wheat growth stages from flowering to maturity (April to May) in the major wheat-producing regions of Hebei Province, China, to collect symptomatic wheat plants infected with FCR. Sampling covered seven prefecture-level cities, including Baoding, Shijiazhuang, Xingtai, Handan, Tangshan, Cangzhou, and Hengshui, representing the principal winter wheat cultivation areas of the province (Figure 1). Within each sampling field, which typically encompassed an area of approximately 1 hectare, a minimum of 5 symptomatic plants were randomly collected. Whole plants, including roots, exhibiting typical FCR symptoms such as stem base necrosis, browning, and whiteheads were carefully uprooted. A zigzag sampling pattern [36] was employed with a minimum interval of 500 m between individual sampling fields. The geographic coordinates (latitude and longitude) of each sampling location were recorded using a handheld GPS device (Kubota, T16, Kubota Corporation, Tokyo, Janpa) to facilitate geospatial analysis.

### 2.2. Fungal Isolation and Culture

Fungal isolation was performed according to a previously described method [37] with minor modifications. Briefly, symptomatic crown/sub-crown tissues were cut into approximately 1 cm segments using sterile scissors, followed by surface sterilization: immersion in 75% ethanol for 40 s, then in 1% sodium hypochlorite for 3 min, and finally rinsing three times with sterile distilled water. The cleaned stem segments were transferred onto sterile filter paper to remove excess moisture and avoid contamination. Using sterile forceps, the dried tissue segments were placed on potato dextrose agar (PDA) medium supplemented with streptomycin (working concentration 0.1 g/L) and incubated in the dark at 25 °C for 3–5 days. For each wheat plant, one tissue segment was selected for fungal isolation. Fungal growth was monitored daily, and colonies emerging from the tissue segments were transferred to fresh PDA plates using sterile toothpicks. Pure cultures of individual strains were obtained through repeated sub-culturing. *Fusarium*-like colonies were subjected to single-spore isolation following the method described in Zhang et al. [38]. The fungal isolates were cultured on PDA medium at 25 °C in the dark using a mold incubator. Purified single-spore isolates were preserved on PDA slants in 1.5 mL tubes and stored at 4 °C for future use. A total of 156 single-spore isolates were obtained in this study and subsequently used for species identification and trichothecene genotype determination.

### 2.3. Genomic DNA Extraction

Fungal isolates were cultured on fresh PDA plates at 25 °C for 3 days in the dark to induce mycelial growth. Total genomic DNA of each fungal strain was extracted from freshly collected mycelia using the CTAB method, as previously described [10]. Briefly, 650 μL of 1× CTAB lysis buffer and approximately 100 μL of quartz sand (CAS: 14808-60-7, Sigma-Aldrich, St. Louis, MO, USA) were pre-added to 2 mL DNA extraction tubes. Aerial mycelia were then collected using sterile toothpicks and transferred into the prepared tubes. Homogenization was performed using a Wonbio-E frozen tissue grinder (Shanghai Wonbio Biotechnology Co., Ltd., Shanghai, China). Subsequent DNA extraction steps followed the protocol described by Zhang et al. [10]. The obtained DNA was finally re-suspended in 100 μL of sterile deionized water, allowed to fully dissolved overnight at room temperature, and then stored at 4 °C. DNA concentration was quantified with the aid of a spectrophotometer, and all samples were diluted to a working concentration of approximately 20 ng/μL with sterile nuclease-free water. Finally, the samples were stored frozen at −20 °C for subsequent PCR applications.

### 2.4. Molecular Confirmation of Fusarium Isolates

All obtained *Fusarium* strains were initially identified based on morphological characteristics, followed by confirmation through molecular detection methods. First, species-specific primers Fp1-1/Fp1-2 (targeting *F. pseudograminearum*) [39] were used for PCR amplification of fungal DNA. The 20 μL PCR reaction system contained of 2 μL of 10× EasyTaq PCR Buffer (TransGen Biotech, Beijing, China), 1 μL of template DNA (20 ng), 0.2 μL of each primer (10 μM), and 16.6 μL of double-distilled water (ddH_2_O). Amplification was performed in a T100 Thermal Cycler (Bio-Rad, Hercules, CA, USA) using the following program: initial denaturation at 95 °C for 3 min; followed by 30 cycles of 95 °C for 30 s, 60 °C for 40 s, and 72 °C for 45 s; with a final extension at 72 °C for 10 min. The amplification products were separated by electrophoresis on a 1.2% agarose gel in TEA buffer, stained with a nucleic acid fluorescent stain (TransGen Biotech, Beijing, China), and visualized under UV light. An expected specific band of 520 bp was amplified from *F. pseudograminearum* isolates. For strains that failed to amplify any band with primer pair Fp1-1/Fp1-2, further amplification was performed using primer pair Fg16F/Fg16R (targeting FGSC) [40] to identify them as members of the FGSC. When necessary, portions of the *translation elongation factor 1-α* (*TEF1*) gene of individual *Fusarium* isolates were amplified using primers EF1/EF2 [41] and bidirectionally sequenced to identify them at the species level through pairwise DNA alignments against the reference database FUSARIOID-ID (https://www.fusarium.org/, accessed on 20 May 2025). All primers used in this study are listed in Table 1. The sequences generated in this study have been deposited in the GenBank database under accession numbers PX578658−PX578677 (Table S1).

### 2.5. Trichothecene Genotype Determination of Fusarium Isolates

To determine the trichothecene chemotype of each *F. pseudograminearum* isolate, a toxin genotype-specific detection method targeting the *Tri8* gene (encoding a key deacetylase enzyme within the core trichothecene biosynthetic cluster) was employed according to Deng et al. [43]. Three specific primer pairs, namely 3AT8-1/3AT8-2, 15AT8-1/15AT8-2, and NIVT8-1/NIVT8-2, were used to identify 3ADON, 15ADON, and NIV genotype *F. pseudograminearum* isolates, respectively. Amplifications were carried out in 20 μL reactions containing 10× EasyTaq PCR Buffer (TransGen Biotech, Beijing, China), specific primers, DNA template, and ddH_2_O, following the methodology described previously [43]. The thermal cycling protocol executed on a Bio-Rad T100 thermal cycler consisted of the following: initial denaturation at 95 °C for 3 min; 30 cycles of 95 °C for 30 s, 60 °C for 30 s, and 72 °C for 50 s; final extension at 72 °C for 6 min; followed by cooling at 12 °C. PCR products were resolved by electrophoresis on 1.2% agarose gels in TAE buffer and visualized under UV light. The expected amplification products for the *Tri8* assay were 424 bp (3ADON), 827 bp (15ADON), and 397 bp (NIV), respectively. The trichothecene genotypes of the FGSC isolates were determined using a previously described PCR-based genotyping protocol targeting the *Tri13* gene [42]. Amplification with primers Tri13P1/Tri13P2 yielded expected fragments of 644 bp for 3ADON, 583 bp for 15ADON, and 859 bp for the NIV genotype (Table 1).

### 2.6. Mating Type Determination of Fusarium pseudograminearum

A previously described, a PCR-based mating type identification assay [44] was employed to determine the mating types of all *F. pseudograminearum* strains in this study. Two mating type-specific primer pairs, fusALPHAfor/fusALPHArev (*MAT1-1* specific, expected amplicon size: 200 bp) and fusHMGfor/fusHMGrev (*MAT1-2* specific, expected amplicon size: 260 bp) (Table 1), were used for the identification of *MAT1-1* and *MAT1-2* strains, respectively. The 20 μL PCR reaction mixture contained 2 μL of 10× EasyTaq PCR Buffer (TransGen Biotech, Beijing, China), 0.2 μL of each primer (10 μM), 1 μL of template DNA (20 ng), and ddH_2_O up to 20 μL. The amplification protocol consisted of an initial denaturation step at 95 °C for 3 min; followed by 30 cycles of denaturation at 95 °C for 30 s, annealing at 60 °C (for fusALPHAfor/fusALPHArev) or 62 °C (for fusHMGfor/fusHMGrev) for 30 s, and extension at 72 °C for 30 s; with a final extension at 72 °C for 6 min. The PCR products were separated by electrophoresis on 1.2% agarose gels and visualized using the staining method described previously. A chi-square test (*p* = 0.05) was applied to assess the deviation of the observed mating type ratio from the expected 1:1 ratio at each sampling site.

### 2.7. Population Genetic Analysis Based on Composite Genotypes

To investigate the population genetic structure of *F. pseudograminearum*, a composite genotype approach was employed. Each isolate was assigned a multi-locus genotype (MLG) by combining its mating type and trichothecene genotype, generating a dataset encompassing two unlinked, functionally important loci. Genetic diversity within each city population was quantified using Nei’s gene diversity index (H_n_) [45] and the Shannon–Wiener index (H_s_) [46]. Nei’s H_n_ estimates the expected heterozygosity under Hardy–Weinberg equilibrium, which is calculated as H_n_ = 1 − Σp_i_^2^, where p_i_ is the frequency of the i-th genotype. The Shannon–Wiener index (H_s_ = −Σp_i_ (lnp_i_)) measures genotypic diversity by incorporating both richness (number of genotypes) and evenness (distribution of genotype frequencies).

## 3. Results

### 3.1. Molecular Identification of Fusarium Isolates

**Figure 2:**
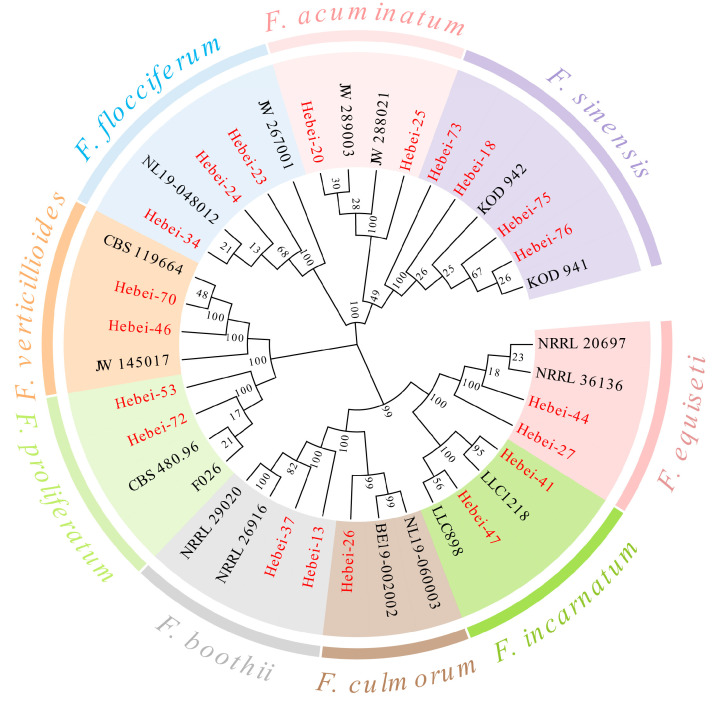
Phylogenetic tree inferred from *TEF1* sequences using the neighbor-joining method in the MEGA11 program. The numbers above internodes represent bootstrap values based on 1000 pseudoreplicates of the data. The isolates obtained in this study are highlighted in red, and the others are reference strains of known species retrieved from the GenBank database (https://www.ncbi.nlm.nih.gov/, accessed on 20 November 2025) (Table S1).

In the current study, a total of 156 fungal isolates were obtained from wheat plants exhibiting typical FCR symptoms (Figure 2). These samples were collected from 46 sampling sites across seven prefecture-level cities in Hebei Province during 2024. All isolates were initially purified by single-spore isolation and subjected to molecular identification using species-specific primer pairs. Initial morphological characterization was conducted based on conidial traits (presence/absence of macroconidia and microconidia, as well as their typical morphologies), mycelia characteristics, and pigmentation, etc., as described in [10,20].

Through a species-specific PCR assay with the primer pair Fp1-1/Fp1-2, 118 isolates yielded a distinct 520 bp amplification fragment characteristic of *F. pseudograminearum*. Subsequently, the remaining 38 isolates that failed to produce the *F. pseudograminearum*-specific amplicon were screened using the FGSC-specific primer pair Fg16F/Fg16R. This assay identified 16 isolates with a 410 bp amplification product and 2 isolates with a 497 bp fragment, confirming them as *F. graminearum* and *F. asiaticum*, respectively.

The majority of the *Fusarium* isolates were thus identified as either *F. pseudograminearum* or members of FGSC. The remaining 20 isolates that did not generate amplicons with either of the aforementioned species-specific primer sets were further characterized by amplification and sequencing of the translation elongation factor *TEF1* gene using primers EF1/EF2. Sequence homology analysis using BLASTN (https://www.fusarium.org/, accessed on 20 May 2025) revealed that these isolates belonged to other *Fusarium* species, including *F. acuminatum* (two isolates), *F. boothii* (two isolates), *F. culmorum* (one isolate), *F. equiseti* (two isolates), *F. flocciferum* (three isolates), *F. incarnatum* (two isolates), *F. proliferatum* (two isolates), *F. sinensis* (four isolates), and *F. verticillioides* (two isolates). The molecular identifications were consistent with the preliminary morphological groupings. Furthermore, molecular evidence derived from partial sequences of the *TEF1* gene provided definitive confirmation of the species identity. All isolates were unambiguously assigned to a species based on high sequence homology with the reference strains in the FUSARIOID-ID database, and this classification was strongly supported by the phylogenetic tree (Figure 2). Collectively, the species identification results confirm that *F. pseudograminearum* is the predominant causal agent of FCR in Hebei Province, accounting for 75.64% of the total isolates, followed by FGSC with 20 isolates (16 *F. graminearum* isolates, 2 *F. asiaticum* isolates, and 2 *F. boothii* isolates).

### 3.2. Trichothecene Genotype Determination

To assess the potential mycotoxin risks associated with the identified *Fusarium* isolates, trichothecene genotyping was performed for all strains. Among the 20 FGSC isolates, PCR amplification using the primer pair Tri13P1/Tri13P2 yielded a specific 583 bp fragment for all isolates, confirming their classification as the 15ADON genotype.

To further clarify the trichothecene chemotypes of the dominant pathogen *F*. *pseudograminearum*, a PCR-based genotyping assay targeting the *Tri8* gene was employed to distinguish between the 3ADON, 15ADON, and NIV genotypes among the 118 identified isolates. The results revealed an absolute dominance of the 15ADON genotype, accounting for 108 strains (91.53%), followed by the 3ADON genotype in 10 strains (8.47%). No NIV genotype was detected in this study. Notably, the number of 15ADON-type strains was significantly higher than that of 3ADON-type isolates.

Geographical distribution analysis revealed that the 15ADON genotype was predominant across all sampling regions in Hebei Province (Table 2). Specifically, the Baoding, Shijiazhuang, and Hengshui populations exclusively exhibited the 15ADON genotype, indicating a highly uniform genotype composition in these areas. In contrast, Xingtai, Handan, Tangshan, and Cangzhou harbored minor populations of 3ADON isolates, among which, Handan had the highest number (four strains). These findings further confirm the overwhelming dominance of the 15ADON genotype in *F. pseudograminearum* populations associated with wheat crown rot in Hebei Province, which is consistent with previous reports from northern China. Additionally, the results indicate relatively low trichothecene genotype diversity within the regional pathogen population.

### 3.3. Mating Type Determination

As a heterothallic species, individual *F. pseudograminearum* isolates harbor only one of the two mating-type idiomorphs (*MAT1-1* or *MAT1-2*) in their genomes, a characteristic that distinguishes it from the homothallic FGSC. Therefore, mating-type analysis was conducted on all 118 *F. pseudograminearum* isolates obtained in the present study.

PCR assays were performed using the mating-type-specific primer pairs fusALPHAfor/fusALPHArev (targeting *MAT1-1*) and fusHMGfor/fusHMGrev (targeting *MAT1-2*). A single, idiomorph-specific band was successfully amplified from each isolate, confirming that all isolates carried one of the two mating-type idiomorphs. Both mating types were identified within the Hebei *F. pseudograminearum* population. Of the 118 *F. pseudograminearum* isolates, 66 isolates (55.93%) were identified as *MAT1-1*, and 52 isolates (44.07%) as *MAT1-2* (Table 3). A chi-square test was performed to evaluate deviations from the expected 1:1 mating-type ratio in the total population. The results showed that the observed *MAT1-1*:*MAT1-2* ratio (1.27:1) did not differ significantly from the expected ratio (χ^2^ = 1.66, *p* = 0.20).

However, the distribution of mating types varied across different sampling locations (Table 3). Notably, the *F. pseudograminearum* population in Cangzhou exhibited a perfectly balanced *MAT1-1*:*MAT1-2* ratio of 1:1. In contrast, other cities showed numerical imbalances: Baoding, Shijiazhuang, Xingtai, Tangshan, and Hengshui had a higher proportion of *MAT1-1*, while Handan had a higher proportion of *MAT1-2*. Nevertheless, chi-square tests revealed that none of these individual regional populations deviated significantly from the expected 1:1 ratio at the *p* = 0.05 significance level. This finding indicates that, despite the observed numerical differences, likely attributed to the limited sample sizes in certain areas, the mating-type ratios of the *F. pseudograminearum* population within each of the seven prefecture-level cities remained in equilibrium.

The overall balanced 1:1 mating-type ratio observed across Hebei Province serves as a key indicator of a potentially sexually competent *F. pseudograminearum* population. This finding suggests that sexual recombination may be occurring or has historically occurred in this region, which could contribute to the genetic diversity and adaptive evolution of the pathogen. Such genetic variability may further have implications for the virulence of *F. pseudograminearum* and its ability to adapt to local agricultural practices.

### 3.4. Population Genetic Diversity Based on Composite Genotypes

Composite genotype analysis provided insights into the population genetic diversity of *F. pseudograminearum* in Hebei Province. Due to the absence of NIV-producing *F. pseudograminearum*, all 118 isolates were categorized into four multi-locus genotypes (MLGs); there was a clear dominance of 15ADON-associated genotypes. The *MAT1-1*/15ADON genotype was the most prevalent (61 isolates, 51.69%), followed by *MAT1-2*/15ADON (47 isolates, 39.83%). In contrast, the two 3ADON-associated genotypes were equally rare: *MAT1-1*/3ADON and *MAT1-2*/3ADON each accounted for five isolates (4.24% of the total).

Genetic diversity indices (Nei’s gene diversity index, H_n_; Shannon–Wiener index, H_s_) were calculated for each city-specific population and the total population (Table 4). The total population exhibited an H_n_ of 0.57 and an H_s_ of 0.98, indicating a moderate-to-high level of genetic diversity. Geographically, the highest genetic diversity was observed in populations from Handan (H_s_ = 1.14, H_n_ = 0.65) and Cangzhou (H_s_ = 1.03, H_n_ = 0.59)—a pattern that correlates with the co-occurrence of both mating types (*MAT1-1* and *MAT1-2*) and both trichothecene genotypes (15ADON and 3ADON) in these regions. In contrast, populations from Baoding, Shijiazhuang, and Hengshui—where the 15ADON trichothecene genotype was fixed—displayed relatively lower diversity. Collectively, the moderate-to-high overall genetic diversity, combined with the previously observed balanced mating-type ratio, supports the existence of a sexually competent *F. pseudograminearum* population in Hebei Province with the potential for genetic recombination.

## 4. Discussion

FCR has emerged as a major threat to wheat production in China, particularly in the water-limited environments of the North China Plain [47]. Hebei Province, a significant winter wheat-growing region, is severely affected. This study presents the first systematic analysis of the species composition of wheat crown rot pathogens, the distributions of trichothecene genotypes, and the mating type structure of *F*. *pseudograminearum* in Hebei Province in 2024. Our results clearly demonstrate that *F. pseudograminearum* is the predominant pathogen, accounting for 75.64% of all isolates, followed by FGSC (12.82%). Within the *F. pseudograminearum* population, the 15ADON genotype was overwhelmingly dominant (91.53%), with only a small proportion (8.47%) being 3ADON producers, while no NIV genotype was detected. Furthermore, the mating type ratio (*MAT1-1*:*MAT1-2*) was nearly 1:1, and population genetic analysis based on composite genotypes revealed a moderate to high level of genetic diversity (H_n_ = 0.57; H_s_ = 0.98). Together, these findings provide strong evidence for a sexually active population with significant potential for genetic recombination and adaptive evolution. These findings are consistent with recent reports from northern China [20,43], including the neighboring Henan Province [10], but contrast with the findings from populations from Australia [48], Canada [49], and southern China [31]. This highlights significant regional and ecological differences in pathogen structure and toxigenic potential.

Since its first detection in China in 2011 [50], wheat FCR caused by *F. pseudograminearum* has spread rapidly and inflicted substantial yield losses in recent years. In recent years, FCR has become highly prevalent across China, particularly in the Huanghuai wheat-growing region, driven by multiple complex factors [10]. It was reported that wheat FCR led to annual grain yield losses exceeding 3.5 billion kg in China in 2024. The escalating incidence and severity of FCR in the Huanghuai wheat-growing region have garnered significant attention from researchers and government authorities. Combining previous surveys with the findings presented herein, it is clear that *F*. *pseudograminearum* is currently the predominant pathogen responsible for FCR in China, with a particular prevalence in warm and arid areas. Compared to a prior survey on wheat FCR in Hebei, the highest prevalence of *F. pseudograminearum* was observed by Mawcha et al. [20] and the present study, suggesting that this pathogen had already become established in the region prior to the initiation of these surveys. Population dynamic analyses of FGSC, the causal agent of FHB of wheat, have revealed shifts toward more aggressive and toxigenic populations in China [51], Norway [52], and the USA [53]. However, whether a similar shift is occurring among the causative agents of FCR in China remains to be evaluated.

Despite their low frequency, several *Fusarium* pathogens other than *F. pseudograminearum* were identified in Hebei Province in this study. In all, 12 *Fusarium* species were identified in this work. Among these pathogens, *F. pseudograminearum* and *F. graminearum* were the predominant species causing FCR of wheat, with 118 (75.64%) and 16 (10.26%) isolates, respectively. The findings of the present study are consistent with previous observations that, except for the dominant pathogen *F. pseudograminearum*, members of *F. acuminatum*, *F. asiaticum*, *F. boothii*, *F. culmorum*, *F. equiseti*, *F. flocciferum*, *F. graminearum*, *F. incarnatum*, *F. proliferatum*, *F. sinensis*, and *F. verticillioides* are also responsible for FCR of wheat in different wheat-growing regions around the world [10,14,15,16,17,18,19,20,21,22,23,24,25,26,27,28,29,30,31,54]. Specifically, all these 11 minor *Fusarium* species were already reported from specific regions of China, including Hebei Province [10,17,18,19,20,21,30,31,54]. However, to our knowledge, this is the first report of *F. boothii*, *F. equiseti*, *F. flocciferum*, *F. incarnatum*, *F. proliferatum*, *F. sinensis*, and *F. verticillioides* causing crown rot of wheat in Hebei Province.

The dominance of *F. pseudograminearum* in Hebei aligns with its adaptation to warm, semi-arid conditions [3,39], which characterize much of the North China Plain. Local agronomic practices, such as wheat–maize rotation with residue retention, combined with severe water scarcity, create favorable conditions for the survival of this residue-borne pathogen [5,30]. As previous reported, *F. pseudograminearum* can survive in infected crop residue for up to three years as fungal hyphae [55,56]. The diseased stubble of the previous crops acts as the main source of the inoculums of *Fusarium* pathogens. Our results confirm a broader shift in the FCR pathogen complex across northern China: from *F. asiaticum* (3ADON) to *F. pseudograminearum* (15ADON). This shift may be attributed to the superior ecological fitness of *F. pseudograminearum* under local agronomic and climatic conditions [57], including enhanced saprophytic competitiveness and the capacity to produce perithecia in drier environments [51]. Notably, the near absence of *F. asiaticum* in our study stands in stark contrast to previous surveys in Hebei [31], highlighting the rapid and dramatic ecological succession occurring within the FCR pathogen complex. Recent work by Mawcha et al. [20] in Hebei also identified *F. pseudograminearum* as the dominant species (91% of isolates), further confirming its prevalence in the region. Their observation of a high detection rate for the DON genotype (84.50%) is consistent with our findings in this present study, reinforcing the stability of this pathogen profile across different sampling periods.

Notably, the 15ADON genotype is nearly fixed within the *F. pseudograminearum* population in Hebei Province. This stands in stark contrast to western Canadian populations, where the 3ADON genotype dominates [49], and Algerian populations, which are predominantly 3ADON-producing *F. culmorum* [25]. However, it aligns with recent findings from Henan and Shandong provinces, highlighting a north–south divergence in trichothecene genotype distribution across China. This geographical pattern may reflect adaptive responses to regional climatic conditions; for instance, 15ADON producers are thought to be better adapted to cooler, drier environments [17], whereas 3ADON and NIV genotypes prevail in warmer, more humid regions. Such environmental adaptation may also explain the absence of NIV genotypes in the present study, which is consistent with reports from other northern Chinese provinces [10,43]. The overwhelming dominance of 15ADON producers raises significant food safety concerns. DON and its acetylated derivatives are stable during processing, can accumulate in grains, and pose substantial risks to human and animal health [11,58]. The present results exacerbate these concerns, as China currently lacks regulatory standards for 15ADON. The high prevalence of toxigenic isolates in Hebei, coupled with the potential for asymptomatic infection and post-harvest toxin accumulation [2], underscores the urgent need for integrated management strategies. These should include the deployment of resistant cultivars, cultural practices such as rotation with non-host crops, and continuous monitoring of pathogen populations and toxin levels [51,59]. On the other hand, the dominance of 15ADON-producing *F. pseudograminearum* in Hebei’s FCR pathogen population contrasts sharply with the prevalence of 3ADON-producing *F. asiaticum* in the region’s FHB pathogen population. This clear niche specialization, both in pathogen species and trichothecene toxin genotype, suggests distinct host–pathogen–environment interactions underlying the two diseases, likely reflecting differences in ecological fitness [60,61]. The precise mechanisms driving this divergence remain to be elucidated.

A key novel aspect of our study is the analysis of the mating-type distribution within the *F. pseudograminearum* population, a parameter not investigated in the prior survey by Mawcha et al. [20]. Our discovery of a nearly 1:1 ratio of *MAT1-1* to *MAT1-2* idiomorphs across Hebei Province provides the first evidence for a sexually competent population of this pathogen in the region. This finding is strongly supported by our population genetic analysis, which revealed moderate-to-high genetic diversity (H_n_ = 0.57; H_s_ = 0.98), a pattern consistent with ongoing genetic recombination. Collectively, these results indicate a high potential for adaptive evolution [44,62]. Such genetic flexibility can accelerate the evolution of traits like fungicide resistance or enhanced virulence, posing a significant challenge for long-term disease management. The overall balanced mating-type ratio aligns with the hypothesis that Hebei may be near the origin of *F. pseudograminearum* in China [30]. However, minor local deviations from the 1:1 ratio in some prefectures warrant future investigation with larger sample sizes. Furthermore, future studies incorporating larger and more balanced samples across different trichothecene genotypes would be valuable for exploring potential associations between toxin production and mating type. Such research could shed light on the evolutionary dynamics of these key traits.

## 5. Conclusions

This study provides a comprehensive analysis of the *Fusarium* species composition, trichothecene genotype distribution, and mating-type structure associated with wheat FCR in Hebei Province, China, during the key growth stages from flowering to maturity (April to May) in 2024. Our results clearly identify *F. pseudograminearum* as the predominant pathogen, accounting for 75.64% of all isolates, with the 15ADON genotype being overwhelmingly dominant (91.53%) within this species. The discovery of a near-balanced mating type ratio (*MAT1-1*:*MAT1-2* ≈ 1:1), coupled with moderate to high genetic diversity (H_n_ = 0.57; H_s_ = 0.98), provides compelling evidence for a sexually active population with significant potential for genetic recombination and adaptive evolution.

These findings reveal a significant shift in the FCR pathogen population across northern China: from the previously reported dominance of *F. asiaticum* (3ADON genotype) to the current predominance of *F. pseudograminearum* (15ADON genotype). This shift is likely driven by regional climate conditions and the widespread adoption of wheat–maize rotation systems with straw retention. The high prevalence of toxigenic strains poses substantial risks to both wheat yield and food safety, primarily due to the potential for DON contamination. Given the genetic diversity and strong adaptive potential of this pathogen population, continuous monitoring and genotype-specific management strategies are imperative. Future research and practical efforts should focus on three key areas: developing resistant wheat varieties, optimizing agronomic practices, and implementing integrated disease management approaches. These measures will be critical to mitigating the impact of FCR and ensuring the sustainable production of wheat in Hebei Province and other ecologically similar regions.

## Figures and Tables

**Table 1:** Primers used in this study.

Target	Primer	Nucleotide Sequence (5′ to 3′) *	Product Size (bp)	Reference
*F. pseudograminearum*	Fp1-1	CGGGGTAGTTTCACATTTCYG	520	[39]
	Fp1-2	GAGAATGTGATGASGACAATA		
FGSC isolates	Fg16F	CTCCGGATATGTTGCGTCAA	400–500	[40]
	Fg16R	GGTAGGTATCCGACATGGCAA		
*TEF1* gene	EF1	ATGGGTAAGGAGGACAAGAC	700	[41]
	EF2	GGAAGTACCAGTGATCATGTT		
Trichothecene genotyping	Tri13P1	CTCSACCGCATCGAAGASTCTC	644, 583	[42]
of FGSC isolates	Tri13P2	GAASGTCGCARGACCTTGTTTC	and 859	
*F. pseudograminearum*-	3AT8-1	CCTTATGACTCCCCCGATGTCG	424	[43]
3ADON	3AT8-2	TGTTTACCACCAGACCGGAC		
*F. pseudograminearum*-	15AT8-1	AAGCGCGCTCATGTCAGTCCAAGTT	827	[43]
15ADON	15AT8-2	GCCCACCGACAGTATTCCTT		
*F. pseudograminearum*-	NIVT8-1	GTACACCGCGAGCGCTATTTCTTCT	397	[43]
NIV	NIVT8-2	CGTGAGACCCAACAGCAT		
*MAT1-1*	fusALPHAfor	CGCCCTCTKAAYGSCTTCATG	200	[44]
	fusALPHArev	GGARTARACYTTAGCAATYAGGGC		
*MAT1-2*	fusHMGfor	CGACCTCCCAAYGCYTACAT	260	[44]
	fusHMGrev	TGGGCGGTACTGGTARTCRGG		

* Degenerate sites underlined indicate the following: K = G or T; R = A or G; S = G or C; Y = C or T.

**Table 2:** Trichothecene genotypes of *Fusarium pseudograminearum* in Hebei, China, in 2024.

Sampling City	Total Number of Isolates	Number and Frequency (%)		
		15ADON	3ADON	NIV
Baoding	5	5 (100.00)	0 (0.00)	0 (0.00)
Shijiazhuang	24	24 (100.00)	0 (0.00)	0 (0.00)
Xingtai	14	13 (92.86)	1 (7.14)	0 (0.00)
Handan	22	18 (81.82)	4 (18.18)	0 (0.00)
Tangshan	18	16 (88.89)	2 (11.11)	0 (0.00)
Cangzhou	28	25 (89.29)	3 (10.71)	0 (0.00)
Hengshui	7	7 (100.00)	0 (0.00)	0 (0.00)
Total	118	108 (91.53)	10 (8.47)	0 (0.00)

**Table 3:** Mating-type data of *Fusarium pseudograminearum* strains isolated from Hebei, China, in 2024.

Sampling City	Total Number of Isolates	Number and Frequency (%)		χ^2^	*p*-Value
		*MAT1-1*	*MAT1-2*		
Baoding	5	3 (60.00)	2 (40.00)	0.20	0.66
Shijiazhuang	24	14 (58.33)	10 (41.67)	0.67	0.41
Xingtai	14	10 (71.43)	4 (28.57)	2.57	0.11
Handan	22	10 (45.45)	12 (54.55)	0.18	0.67
Tangshan	18	11 (61.11)	7 (38.89)	0.89	0.35
Cangzhou	28	14 (50.00)	14 (50.00)	0.00	1.00
Hengshui	7	4 (57.14)	3 (42.86)	0.14	0.71
Total	118	66 (55.93)	52 (44.07)	1.66	0.20

**Table 4:** Composite genotype analysis and genetic diversity assessment of *Fusarium pseudograminearum* populations in Hebei Province.

Sampling City	Total Number of Isolates	*MAT1-1*/15ADON	*MAT1-2*/15ADON	*MAT1-1*/3ADON	*MAT1-2*/3ADON	Nei’s Gene Diversity (H_n_)	Shannon–Wiener Index (H_s_)
Baoding	5	3	2	0	0	0.48	0.67
Shijiazhuang	24	14	10	0	0	0.49	0.68
Xingtai	14	10	3	0	1	0.40	0.76
Handan	22	9	9	1	3	0.65	1.14
Tangshan	18	9	7	2	0	0.59	0.96
Cangzhou	28	12	13	2	1	0.59	1.03
Hengshui	7	4	3	0	0	0.49	0.68
Total	118	61	47	5	5	0.57	0.98

## Data Availability

The original contributions presented in this study are included in the article/Supplementary Materials. Further inquiries can be directed to the corresponding author.

## Supplementary Materials

The following supporting information can be downloaded at: https://www.mdpi.com/article/10.3390/jof11120844/s1, Table S1: *Fusarium* isolates used in the phylogenetic analysis and the GenBank accession numbers for their *translation elongation factor 1-α* sequences.

## Abbreviations

The following abbreviations are used in this manuscript:

DON	deoxynivalenol
NIV	nivalenol
3ADON	3-Acetyl-Deoxynivalenol
15ADON	15-Acetyl-Deoxynivalenol
FGSC	*Fusarium graminearum* species complex
FCR	Fusarium crown rot
FHB	Fusarium head blight
