# Supplementary material for: Isolation, Toxigenic Potential, and Mating Type of Fusarium pseudograminearum Causing Wheat Crown Rot in Hebei, China
Source: J Fungi (Basel). 2025 Nov 28;11(12):844. doi: 10.3390/jof11120844 (PMC12733621; doi:10.3390/jof11120844)
Supplement: Supplementary file 1 [file jof-11-00844-s001.zip › jof-3984949-supplementary.pdf]

Table S1. *Fusarium* isolates used in the phylogenetic analysis and their GenBank accession numbers of *translation elongation factor 1-α*.

Species	Strain code	GenBank accession no. *
Fusarium acuminatum	Hebei-20	PX578658
Fusarium acuminatum	Hebei-25	PX578659
Fusarium acuminatum	JW 288021	MZ921907
Fusarium acuminatum	JW 289003	MZ921908
Fusarium boothii	Hebei-13	PX578660
Fusarium boothii	Hebei-37	PX578661
Fusarium boothii	NRRL 26916	GQ915503
Fusarium boothii	NRRL 29020	AF212443
Fusarium culmorum	Hebei-26	PX578662
Fusarium culmorum	NL19-060003	MZ921902
Fusarium culmorum	BE19-002002	MZ921899
Fusarium equiseti	Hebei-27	PX578663
Fusarium equiseti	Hebei-44	PX578664
Fusarium equiseti	NRRL 20697	GQ505594
Fusarium equiseti	NRRL 36136	GQ505644
Fusarium flocciferum	Hebei-23	PX578665
Fusarium flocciferum	Hebei-24	PX578666
Fusarium flocciferum	Hebei-34	PX578667
Fusarium flocciferum	JW 267001	MZ921914
Fusarium flocciferum	NL19-048012	MZ921915
Fusarium incarnatum	Hebei-41	PX578668
Fusarium incarnatum	Hebei-47	PX578669
Fusarium incarnatum	LLC1218	OP487162
Fusarium incarnatum	LLC898	OP487174
Fusarium proliferatum	Hebei-53	PX578670
Fusarium proliferatum	Hebei-72	PX578671
Fusarium proliferatum	F026	MZ399213
Fusarium proliferatum	CBS 480.96	MN534059
Fusarium sinensis	Hebei-18	PX578672
Fusarium sinensis	Hebei-73	PX578673
Fusarium sinensis	Hebei-75	PX578674
Fusarium sinensis	Hebei-76	PX578675
Fusarium sinensis	KOD 942	OL772796
Fusarium sinensis	KOD 941	OL772795
Fusarium verticillioides	Hebei-46	PX578676
Fusarium verticillioides	Hebei-70	PX578677
Fusarium verticillioides	JW 145017	MZ921825
Fusarium verticillioides	CBS 119664	MW401981

* https://www.ncbi.nlm.nih.gov/, accessed on 20 November 2025.
